# Multiple cases of osteopetrosis-like disease in young broiler flocks in Germany

**DOI:** 10.1016/j.psj.2026.107029

**Published:** 2026-04-30

**Authors:** Hicham Sid, Benjamin Schade, Brigitte Böhm, Eva Kappe, Werner Heering, Andreas Brühschwein, Dörte Lüschow, Ferdinand Schmitt

**Affiliations:** a Reproductive Biotechnology, TUM School of Life Sciences Weihenstephan, Technical University of Munich, Freising, Germany; b Bavarian Animal Health Service, Poing, Germany; c Tierarztpraxis Isenthal, St. Wolfgang, Germany; d Small Animal Clinic of the LMU, Centre of Veterinary Clinical Medicine, Veterinary Faculty, LMU Munich, Germany; e Division for Poultry, Farm Animal Clinic, School of Veterinary Medicine, Freie Universität Berlin, Berlin, Germany

**Keywords:** Osteopetrosis-like disease, Broiler, Avian Leukosis virus, Genetic factors

## Abstract

Osteopetrosis in chickens is characterized by abnormal osteoblast proliferation and is often caused by avian leukosis virus (ALV). In this study, we report a case of osteopetrosis-like disease in slow-growing broilers, which presented with lameness between days 6 and 17 after hatch. While selection rates of sick birds varied among affected flocks, the animals exhibited similar symptoms, including weakness, swollen legs, and paralysis. Post-mortem investigations primarily revealed moderate to severe symmetrical thickening of the long bones. The histology indicated a diffuse periosteal hyperplasia of the cortical area associated with a pronounced formation of mineralized bone tissue. Given the young age of the affected birds and the lack of detectable exogenous ALV, it is important to explore possible genetic factors contributing to this condition. Moreover, we cannot overlook the possibility that this condition was due to vaccines contaminated with the leukosis virus. Collaborative research is essential to identify any infectious or genetic contributors to this issue.

## Introduction

Over the past fifty years, the poultry industry has experienced significant growth. Modern poultry breeding has boosted meat and egg productivity, but selection for rapid growth and high laying rates has caused welfare problems such as lameness, skeletal disorders, and behavioral issues like feather pecking (Bokkers and Koene, 2003; Julian, 1998; Siegel et al., 2019; Wideman Jr, et al., 2013). One key factor in achieving optimal performance in poultry flocks is maintaining skeletal health. The high demand for rapid weight gain in broilers often leads to bone disorders, resulting in substantial economic losses (Kestin, et al., 2001; Rath, et al., 2000; Świątkiewicz and Arczewska-Wlosek, 2012). This significant weight gain over a short period is not aligned with skeletal development, increasing the risk of deformities and fractures (Julian, 1998). Bone integrity can be influenced by various factors, including age, genetic predisposition, and infectious diseases. Additionally, nutritional deficiencies of calcium, phosphorus, or vitamin D may lead to diminished skeletal growth and altered mineralization (Rath, et al., 2000; Wilson and Duff, 1991). Infectious agents can also threaten bone quality and are often associated with multifactorial causes, which complicates diagnosis and appropriate treatment (Raehtz, et al., 2018).

Osteopetrosis is a unique skeletal condition that can be caused by avian leukosis virus (ALV), leading to abnormal osteoblast proliferation, particularly in long bones (Brothwell, 2002; Robinson, et al., 1986). There are 11 subgroups of ALV, named A to K. Among these, ALV-E, ALV-F, and ALV-G are endogenous, while the others are exogenous and may lead to mortality rates ranging between 1 and 2% in Galliformes (Fandiño et al., 2023). ALV subgroups are classified based on host range, the gp85(SU) envelope protein sequence, receptor usage in susceptible vs. resistant bird cells, and genomic features (including whether viruses are exogenous or endogenous) (Fandiño et al., 2023). The ability of ALV, particularly subgroup J (ALV-J), to cause osteopetrosis is related to its neoplastic nature, which is also responsible for myeloblastosis, myelocytomatosis, hemangiomas, and erythroblastosis (Fadly, 2000; Fandiño et al., 2023). ALV is known to induce osteopetrosis at a low frequency after a long latent period (Payne, 1998; Schmidt and Smith, 1982). Viral strains, such as myeloblastosis-associated virus type 2 (MAV-2), can cause osteopetrosis within the first few weeks of life, as first reported in the early 1980s (Schmidt and Smith, 1982). This rapid onset of the disease is caused by viral integration, which triggers a localized 'metabolic storm' of osteoblastic activity (Smith and Ivanyi, 1980). This is characterized by an exponential increase in periosteal osteoblastic activity that outpaces natural resorption, leading to replacement of the medullary canal with dense, disorganized woven bone (Smith and Ivanyi, 1980). Genetic associated osteopetrosis in vertebrates including humans and birds is pathogenetically characterized by a defect in osteoclastic bone resorption (Piret, et al., 2016). Moreover, some genetic factors can also lead to osteopetrosis lesions in some Galliformes, including the mutation of the Microphthalmia-associated transcription factor (*Mitf*), which has been identified as a potential factor for osteopetrosis in quails (Kawaguchi et al., 2001; Minvielle et al., 2010). Further mutations have been described in other species, such as humans, but have never been investigated in birds (Leite et al., 2023; Piret et al., 2016). The clinical manifestation is accompanied by a marked concentric thickening of the diaphysis, particularly in the tarsometatarsus, giving the legs a characteristic 'bowed' or 'thick-shanked' appearance (Brothwell, 2002). The transition from normal cortical bone to hyperplastic woven bone indicates a complete loss of homeostatic signaling, where viral integration leads to uncontrolled osteoblastic activity that compromises the medullary cavity (Thorp 1994). These skeletal lesions represent a significant economic burden, resulting in poor feed conversion and increased culling rates, making the management of this condition a priority for maintaining flock productivity (Liu et al., 2023).

In this study, we report osteopetrosis-like disease in slow-growing broilers within the first days after hatching. The common clinical signs in all flocks were lameness, heterogeneous growth, and symmetrical thickening of long bones.

## Materials and methods

### Case history

A total of nine broiler flocks were investigated from November 2019 to October 2021 after exhibiting problems including lameness, paralysis, reduced water and feed intake, and heterogeneous growth based on body weight. Field veterinarians were consulted to obtain detailed information regarding the vaccination protocols implemented for both the chicks and their parent flocks.

All symptoms observed by the clinical teams were documented, and birds were selected for postmortem and laboratory examinations. Young birds of two different genetic backgrounds were affected: Ross 308 (four flocks) and Hubbard Cy 57 (five flocks) (Table 1). The animals originated from four hatcheries, three in Southern Germany and one in Northern Germany (Table 1). In cases 1-5, farms acquired chicks from a single hatchery located in Southern Germany. The size of the investigated flocks ranged from 400 to 30,000 birds, which were housed on deep litter. In three poultry flocks, the selection rate of sick birds that were culled reached 30% within the first seven days of age, whereas in other flocks, it did not exceed 3%. In one farm, all animals had to be culled after consultation with the responsible veterinary office (case #8, Table 1). Birds were chosen for culling based on clinical signs observed during the farmer's daily flock inspections. Specifically, individuals showing severe lameness—such as an inability to walk or those moving on their wings—were identified as diseased. Due to the unfavorable prognosis and animal welfare aspects, these birds were euthanized. Euthanasia was carried out on-site by farmers or field veterinarians. Birds were first rendered immediately unconscious by blunt force trauma to the skull, then killed by cervical dislocation to ensure death. Procedures were performed promptly and without prolonged distress, in accordance with German animal welfare requirements (https://www.gesetze-im-internet.de/tierschg/).

**Table 1:** Summary of broiler farms with cases of osteopetrosis.

Case no.	Farm^1^	Hatching	Age (d)^2^	Number of examined birds^3^	Hatchery	Genetic background
1	A	10.08.2019	17	2	⁎⁎	Hubbard Cy57
2	B	26.10.2019	15 + 24	7 + 4	⁎⁎	Hubbard Cy57
3	C	14.12.2019	13 + 31	3 + 2	⁎⁎	Hubbard Cy57
4	A	15.02.2020	12	5	⁎⁎	Hubbard Cy57
5	D	27.11.2020	12	10	⁎⁎	Aviagen Ross 308
6	E	19.02.2021	6	5	⁎⁎⁎	Aviagen Ross 308
7	F	12.07.2021	10	3	*	Aviagen Ross 308
8	G	Unknown	Unknown	4	⁎⁎⁎⁎	Aviagen Ross 308
9	F	14.10.2021	Unknown	4	*	Hubbard Cy57

1 Farms indicate a single-age flock with osteopetrosis-like lesions.

2 Age of the examined flock. Two flocks were visited twice (cases no. 2 and 3).

3 Number of necropsied birds each visit. Birds in two flocks were necropsied twice (cases no. 2 and 3).

⁎ Birds originated from a shared hatchery in South Germany.

⁎⁎ Birds originated from a shared hatchery in South Germany.

⁎⁎⁎ Birds originated from a hatchery in North Germany.

⁎⁎⁎⁎ Birds originated from a hatchery in North Germany.

Organic broilers (Hubbard CY57) from farms A and B received vaccinations on their first day of life against several diseases, including infectious bronchitis (IB), infectious bursal disease (IBD), coccidiosis, and Marek's disease. Marek's vaccination was administered by injection with a Rispens strain combined with an HVT-IBD vector vaccine. The IB and coccidiosis vaccines were applied by spray. In contrast, conventional broilers (Ross 308) from farms C and D were only vaccinated against infectious bronchitis (IB) on their first day of life, using the spray method. No information on the vaccination status of the parental flocks (Hubbard CY57) was available, as they were raised in the Netherlands or France and purchased as adult birds by a German poultry company.

### Postmortem analysis

A total of 49 broiler chickens were necropsied according to standard protocols. Tissue samples from the heart, liver, spleen, kidney, intestine, brain, and affected long bones were collected for histology. Furthermore, bone, kidney, and liver samples were collected for PCR analysis. Cloacal swabs were used for antigen-ELISA.

### Histology

Tissue samples were fixed in 10% neutral buffered formalin and embedded in paraffin blocks. Tissue sections 4 µm thick were mounted on glass slides and stained with hematoxylin and eosin (H&E).

### Radiography

Radiographic examinations of the limbs were performed using an Axiom Luminos dRF Max system equipped with a Fluorospot Compact FD flat-panel detector (VA01A; Siemens Healthineers, Erlangen, Germany). Standard projections included mediolateral and craniocaudal views of each wing and pelvic limb. Exposure parameters were 48 kVp, 14 mAs, and a source-to-detector distance of 115 cm. No grid was used. The imager pixel spacing was 0.148 × 0.148 mm.

### ELISA

The enzyme-linked immunosorbent assay (ELISA) was performed to detect the p27 antigen, which is present in all ALV subgroups, including both exogenous and endogenous ALV subgroups. This was performed using the IDEXX ALV Ag Test, as per the manufacturer’s instructions (IDEXX Laboratories, Inc., USA).

### DNA extraction

Automated total DNA extraction from tissue material was conducted using the QIAamp DNA Mini QIAcube Kit® (Qiagen GmbH, Hilden, Germany) according to the manufacturer’s instructions. Analyzed organs included kidneys, bone, and liver tissues.

### PCR

For screening exogenous ALV in bone, liver, and kidney tissues, comprising subgroups A-D and J, we used a nested PCR with two primer pairs, as described elsewhere (García et al., 2003). Both pairs were located within the long terminal repeat region and amplified fragments within a range from 350 bp to 318 bp (primer pair Leu3.2F/Leu7R) and 175 bp to 220 bp (Leu11F/ Leu12R), respectively. The first PCR was conducted with the Taq PCR Master Mix Kit (Qiagen) by adding 12.5 µl Taq PCR Master Mix, 12.5 pmol of each primer (Leu3.2F/Leu7R), and nuclease-free water to a final volume of 25 µl. The second PCR was performed similarly, using the primer pair Leu11F/Leu12R and 2.5 µl of the PCR product from the first PCR as template. The cycling conditions for the first PCR were an initial step of 94°C for 4 min followed by 30 cycles of denaturation at 94°C for 20 sec, annealing at 60°C for 40 sec, and extension at 72°C for 1 min, followed by a final extension step at 72°C for 2 min. The conditions for the second PCR were the same, but with a total of 35 cycles. PCR products were analyzed by electrophoresis in 1.5% and 2% agarose gels, respectively, and stained with ethidium bromide.

In a second approach, we conducted a broad spectrum (BS) PCR with subsequent sequence analysis using the primer pair BS-UP and BS-DWN (Spencer et al., 2003). This should lead to amplification of a 1491 bp fragment of the envelope gene in all chicken relevant subgroups of ALV, RSV, and MAV, except subgroup J strains. BS-PCR was carried out with the Q5® Hot Start High-Fidelity 2X Master Mix (New England Biolabs GmbH, Frankfurt am Main, Germany) in a total volume of 25 µl containing 12.5 µl Q5 High-Fidelity 2X Master Mix, 12,5 pmol of each primer, and up to 500 ng DNA. Samples were subjected to an initial step of 98°C for 30 sec, followed by 35 cycles consisting of 1 min denaturation at 98°C, 30 sec annealing at 60°C, and 1,5 min extension at 72°C. A final extension step was performed for 2 min at 72°C. PCR products were analyzed by 0.8% agarose gel electrophoresis. After electrophoresis, fragments of the expected size of about 1500 bp were gel-purified using the Monarch® DNA Gel Extraction Kit according to the manufacturer’s recommendations (New England Biolabs) and sequenced directly using the primer BS-UP by a commercial sequencing service (LGC Genomics GmbH, Berlin, Germany). Positive controls based on known ALV-positive DNA and negative controls (nuclease-free water) were used for all PCR runs.

### Phylogenetic analysis

The nucleotide sequences were analyzed with BASIC BLAST program to search for sequence similarities with previously reported envelope gene sequences of ALV found on GenBank. The aligned sequences were subjected to bootstrap (1000 replicates), and the bootstrap-generated data sets were used to construct the phylogenetic tree by the Maximum Likelihood method and the Tamura-Nei model implemented in MEGA version 11 (Tamura et al., 2021).

## Results

### Clinical examination revealed osteopetrosis-like disease

**Fig. 1:**
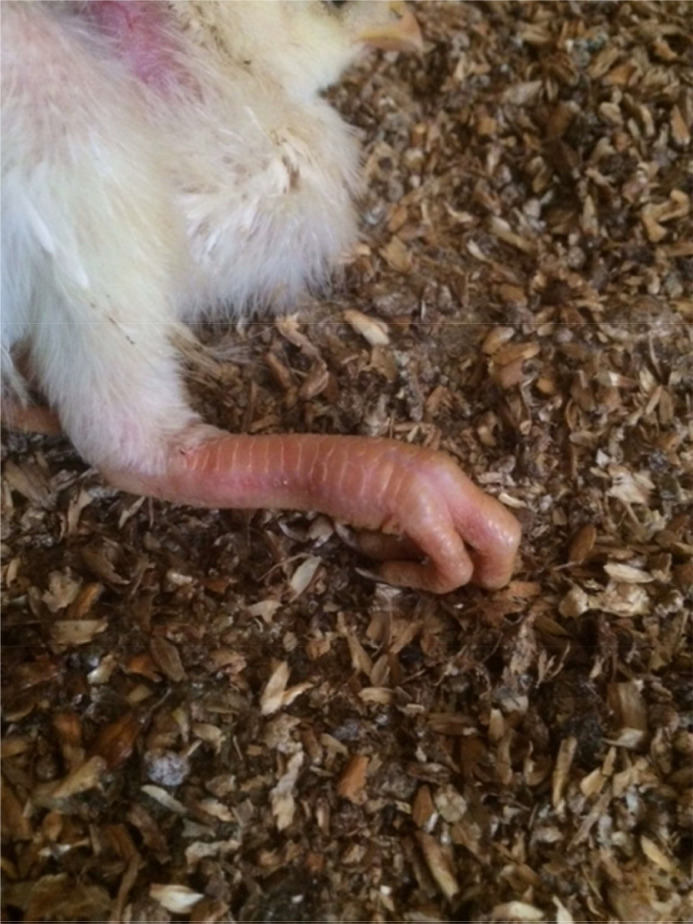
Clinical picture of an eleven-day-old broiler chicken suffering from osteopetrosis associated with reddened skin of the legs, lameness, and thickening of the long bones, resulting in difficulty accessing feed and water.

The clinical signs appeared between days 6 and 17 after hatch, except in one flock that was investigated at two months of age and suffered from a recurring case of osteopetrosis (Fig. 1). Clinical signs were heterogeneous growth, paralysis, and lameness, resulting in difficulty in accessing feed and water (Table 1).

Despite extensive inquiries, no detailed vaccination history, particularly those administered in the hatchery on the first day of life, could be retrieved for the breeder flocks. During the rearing period, the breeder chicks received a vaccination program commonly used in Germany and Austria, consisting of standard live (drinking-water) vaccines and inactivated vaccines. All organic broiler chicks were vaccinated with a turkey herpesvirus (HVT)-based vector vaccine carrying a gene cassette encoding the VP1 antigen of infectious bursal disease virus (IBDV) and a Marek’s disease (Rispens CVI-988). In contrast, conventionally reared broilers (Ross 308) received only live vaccines against infectious bronchitis (multiple strains), Newcastle disease, and infectious bursal disease.

### Predominant osteopetrosis lesions in long bones

**Fig. 2:**
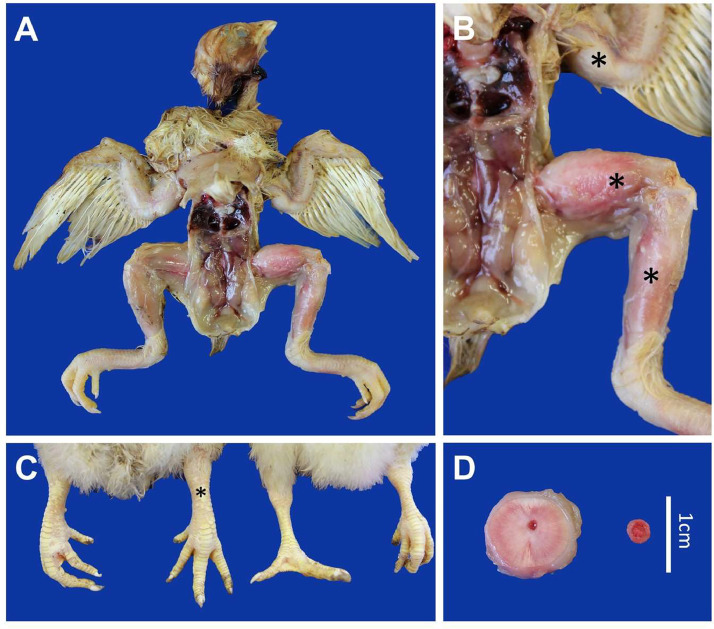
Macroscopical pictures of osteopetrosis-like lesions of 10-day-old broilers. A) Overview B) thickened humerus, femur, and tibiotarsus marked with asterisks. C) thickened tarsometatarsus (left bird) compared to a healthy control animal (right). D) transversal section of the affected femur (left) of approximately 1 cm thick compared to a healthy control femur (right) of approximately 0.35 cm thick. While this condition is often bilateral, severity can differ between individual bones.

Of the 49 examined broilers, 40 showed moderate to severe thickening of the diaphysis of long bones. The external diameter of the affected long-bone diaphysis was 2-3 times the diameter of birds of the same age. The periosteum could not be easily separated from the bone. Femur, tibiotarsus, and tarsometatarsus were most frequently affected. In severely affected cases, the femur had a diameter exceeding one centimeter (Fig. 2). Other bones, like the radius, ulna, skull, and vertebral column, were less affected. Additionally, 16 out of 49 birds 32%) exhibited moderate kidney swelling, while the other organs appeared normal. However, it is crucial to mention that kidney-to-body weight ratios were not recorded in this routine field pathology setting, which represents a limitation of this field-based study.

### Analysis of bone and renal tissue: cortical thickening and nephropathy

**Fig. 3:**
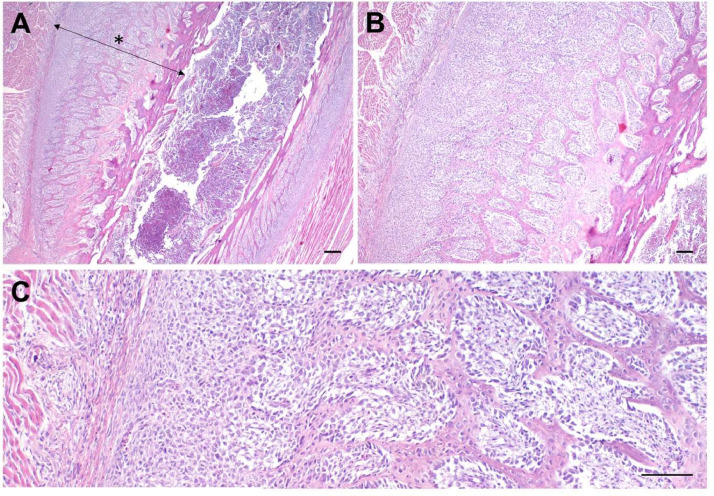
Histological pictures of osteopetrotic femur from a clinically sick animal at eleven days after hatching. The pictures show a longitudinal section of the diaphysis. There is a severe proliferation of osteoblasts in the periphery, followed by a development of woven bone towards the substantia compacta (asterix). Hematoxylin-eosin (HE) stain; A: 20x, B: 40x, C:100x, respectively; Scale bars A: 200 μm, B: 100 μm, and C: 100 μm.

Bone histology revealed a pronounced, diffuse, and mostly circumferential thickening of the cortical bone tissue. There was a marked proliferation of periosteal osteoblasts followed by the formation of osteoid and immature, woven bone (Fig. 3). Ossteoclasts were present on the endosteal surface. Epiphyseal growth plates were not affected. In some cases, the width of the medullary cavity decreased due to an increase in bone tissue. The swollen kidneys exhibited acute tubular epithelial degeneration, multifocal mineralization, and intratubular urate deposition with a granulomatous reaction.

### Radiographic confirmation of the skeletal pathology

**Fig. 4:**
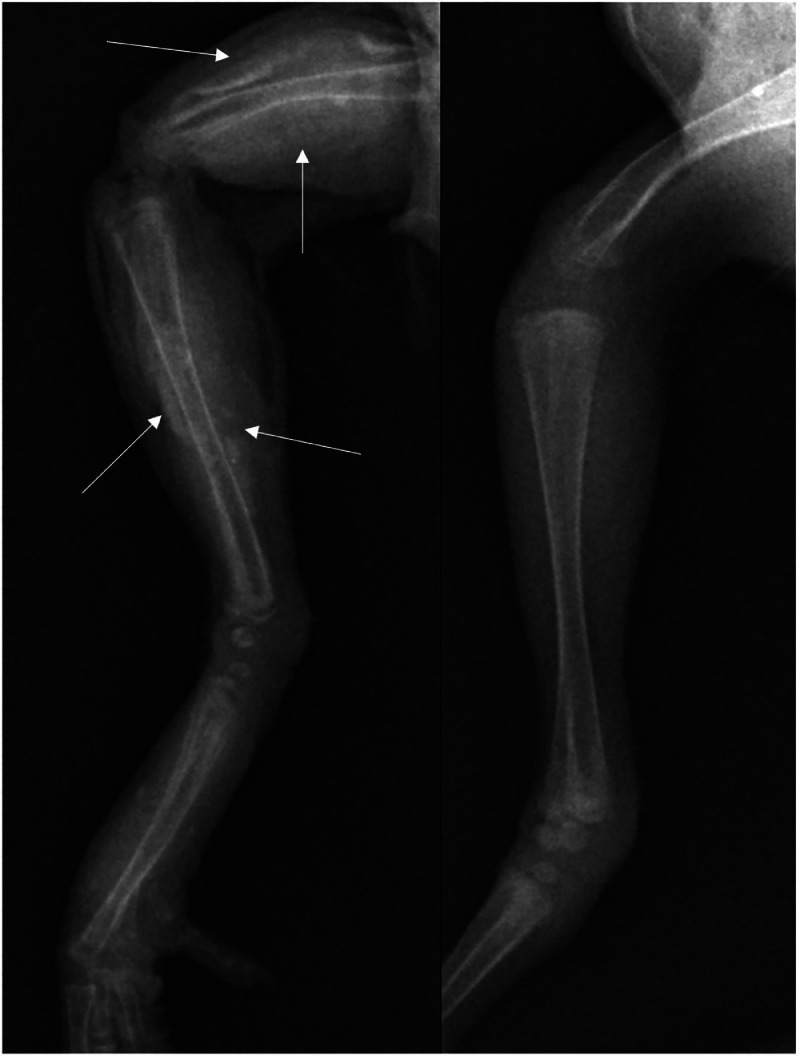
X-ray comparison of the right hindlimbs of a healthy and an osteopetrotic broiler. Left: severely affected broiler with diaphyseal, cloudy to lamellar mineralization and amorphous new bone formation on femur, tibiotarsus, and tarsometatarsus. Arrows indicate the areas of amorphous new bone formation and diaphyseal mineralization. Right: control bird.

For a radiographic conformation of the skeletal pathology, two representative10-day old broilers of Farm F (Table 1) were investigated, one severely affected and one unaffected (control) bird. In the control animal, the cortex of all long bones of the wing and pelvic limb appeared fine, sharply delineated, and regularly mineralized, with a clearly defined corticomedullary junction. In the severely affected broiler, there was a severe, bilaterally symmetrical pattern of diaphyseal, cloudy to lamellar mineralization and amorphous new bone formation, poorly defined and surrounding the cortices of all long bones, most severely in the humerus and femur (Fig. 4). In both animals, the joint spaces were widened, consistent with age-appropriate incomplete ossification of the epiphyses and the carpal and tarsal bones.

### Antigen ELISA indicated the possible involvement of ALV

We used ELISA to provide an add-on to the previous laboratory investigations. A total of 20 cloacal swab samples were collected from a later case with osteoperotic-like lesions (year 2023, not shown in Table 1) and analyzed to detect p27 protein, which is shared among all ALV subtypes. The results showed that 4 out of 20 samples were ALV-positive (Supplementary data).

### No exogenous ALV detection in the tissues

**Fig. 5:**
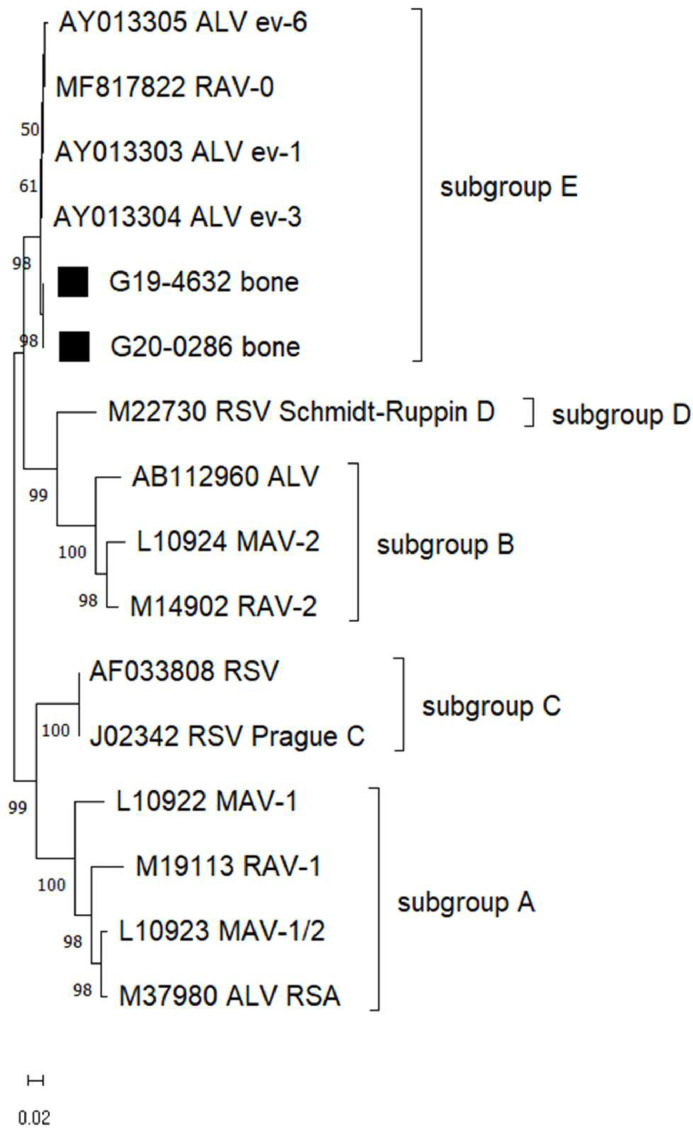
Phylogenetic analysis of the partial nucleic acid sequence (848 bp) of the envelope protein gene of ALV. Data were analyzed using the maximum likelihood method. Sequences from affected flocks are marked with black squares. For comparison, GenBank sequences of representatives of the various ALV subgroups were included.

All investigated tissues were negative for exogenous ALV (A, B, C, D, J) in the nested PCR. In a second approach using the BS-PCR, fragments of the expected size were amplified in all four samples. BLAST analysis of the obtained partial envelope gene sequences revealed the closest matches with endogenous ALV. Further phylogenetic analysis confirmed the presence of endogenous ALV subgroup E (Table 2 and Fig. 5). The analyzed sequences clustered with endogenous ALV.

**Table 2:** PCR screening of ALV in flocks with osteopetrosis-like lesions.

Flock no.	Material	Investigation for ALV proviral DNA			
		Exogenous ALV		ALV-BS	
		1. PCR round	2. PCR round	PCR	Sequence analysis
Farm B	Bone	Negative	negative	positive	subgroup E
Farm C	Bone	Negative	negative	positive	subgroup E
	Liver	Negative	negative	positive	subgroup E
	Kidney	Negative	negative	positive	subgroup E

## Discussion

ALV-induced osteopetrosis in chickens typically develops after a latent ALV infection, making this disease apparent in older birds (Schmidt and Smith, 1982). In contrast, this report describes cases of osteopetrosis-like disease in slow-growing young broilers within the first days of age. It is important to note that we reported this condition only in two breeds, Ross 308 and Hubbard C57, which are dominant in Bavaria, and that osteopetrosis was not observed in other broiler breeds, such as Cobb Sasso and Ranger, which are underrepresented in the studied region. The animals exhibited clinical symptoms and histological lesions typically associated with MAV-2 (O) strain-induced lesions after *in ovo* infection (Powers et al., 1987). MAV-2 (O) can cause rapid-onset osteopetrotic lesions after *in ovo* injection into 19-day-old chicken embryos, leading to bone lesions as early as seven days post-hatch (Powers et al., 1987). Interestingly, injection of 8-day-old chickens with the MAV-2 (O) strain resulted in 100% anemia without signs of osteopetrosis (Paterson and Smith, 1978).

The clinical disease of osteopetrosis caused by ALV was previously attributed to exogenous virus subtypes due to the congenital transmission of the virus (Smith and Ivanyi, 1980; Wang et al., 2020). Surprisingly, in the present case, the standard PCRs were negative for all ALV-exogenous subtypes. New strains may emerge that could evade detection by standard laboratory methods (Nishiura et al., 2020). The vertical transmission of ALV-J from infected breeding flocks can lead to immunosuppression in offspring and even significantly reduce the efficacy of the Marek’s disease vaccine, as indicated by a low vaccine protective index (Wang et al., 2020). Cases of ALV-J-induced osteopetrosis have been reported in adult birds, including broilers aged 5 to 7 weeks, which exhibited osteopetrosis characterized by thickening and lumen narrowing of the sternum, with dentation of the periosteum (Fotouh et al., 2024).

We hypothesize that ALV infection in offspring could be associated with hatchery-origin transmission, since many of the affected broiler flocks originated from the same hatchery (Table 1). Furthermore, the possibility of vertical transmission of ALV cannot be excluded, especially since previous studies have indicated that contaminated live vaccines may be a source of the disease in offspring (Mao et al., 2020). In our case, the incomplete vaccination history of the breeder flocks precluded any assessment of whether a contamination with avian leukosis viruses may have originated from a vaccine. Organic broiler chicks were vaccinated with a turkey herpesvirus (HVT)-based vector vaccine from one manufacturer and a Rispens CVI-988- based vaccine from another manufacturer. However, vaccine batch numbers were unavailable and therefore could not be compared. This is a relevant limitation because vaccine-associated contamination—when it occurs—is often limited to a small number of batches and may affect distribution for a limited period (Mao et al., 2020).

The possibility of a contaminated vaccine is not supported by the fact that Ross 308 broilers were also affected. In Germany, this genetic line is generally not vaccinated against Marek’s disease, meaning they did not receive either the HVT vector vaccine or a live Marek’s disease vaccine. Despite this, these birds still developed a similar clinical picture, albeit with fewer cases.

In most affected flocks, sick animals had to be selected immediately after showing an inability to walk, which limited additional sampling. Further symptoms or histological lesions, such as anemia or nephroblastoma, were not observed.

Besides ALV-induced osteopetrosis, genetic disorders have been reported to cause this condition in various species, including birds and humans. Previous studies in other Galliformes, such as quail, have shown that homozygous mutation of the Microphthalmia-associated transcription factor (*Mitf*) gene leads to dysfunction in osteoclastogenesis (Kawaguchi et al., 2001; Minvielle et al., 2010). Authors reported osteopetrotic phenotype in one-month-old quails, which exhibited high radiopacity at the epiphyseal-metaphysis region of long bones (Kawaguchi et al., 2001). Moreover, human cases of autosomal recessive osteopetrosis in infants, most known as malignant infantile osteopetrosis, were reported at an incidence of about 1 in 250,000 live births (Amirfiroozy et al., 2025). Furthermore, a few reports in humans described osteopetrosis associated with renal tubular acidosis (Piret et al., 2016) which may lead to nephrocalcinosis (Sly et al., 1991). While Osteopetrosis associated with renal tubular acidosis is caused by a genetic defect in the chloride/proton antiporter 7 (*CLCN7*) (Piret et al., 2016), osteopetrosis leading to nephrocalcinosis is due to a homozygous mutation in carbonic anhydrase 2 (*CA2*) (Sly et al., 1991). There are major genetic differences between humans and birds; although the genetic defects documented in humans were not reported in chickens, investigating potential genetic bone diseases in birds would be worthwhile. Since the affected broilers originated from different genetic strains, a genetic cause of this condition in the investigated poultry flocks seems unlikely. Interestingly, infant cynomolgus monkeys developed osteopetrosis after *in utero* exposure to a human IgG2 monoclonal antibody that binds and inhibits *RANKL* activity (Boyce et al., 2014). Additional studies also described cases of drug-induced osteopetrosis, like repeated administration of high doses of the aminobisphosphonate zoledronic acid, usually prescribed for children with osteogenesis imperfecta (Whyte et al., 2023).

Nutritional causes, such as a calcium-phosphorus imbalance (Chen and Moran Jr, 1995) or secondary renal hyperparathyroidism, can lead to impaired bone structure and affect mineral metabolism (Naveh-Many and Volovelsky, 2020). Moreover, bone structure revealed no signs of rickets, which was supported by the fact that these flocks were supplemented with a complex of vitamins A, D3, K, as well as calcium-phosphorus complexes. Since different flocks from various hatcheries were affected, a drug-induced osteopetrosis or a nutritional cause seems unlikely.

This report describes osteopetrosis-like disease without a clear indication of the causative agent, which we found to be a real problem in young broilers. The early onset of disease in broiler chicks may be responsible for heterogeneous growth, immunosuppression, and reduced vaccination efficacy. Therefore, it is essential to raise awareness of this disease and to promote collaborative research to identify potential infectious or genetic factors that contribute to its development.

## Funding

Parts of this work were financially supported by the Free State of Bavaria and the Bavarian Joint Founding Scheme for the Control and Eradication of Contagious Livestock Diseases.

## CRediT authorship contribution statement

**Hicham Sid:** Writing – review & editing, Writing – original draft, Project administration, Conceptualization. **Benjamin Schade:** Writing – review & editing, Writing – original draft, Validation, Methodology, Investigation, Formal analysis, Conceptualization. **Brigitte Böhm:** Writing – original draft, Methodology, Formal analysis, Data curation, Conceptualization. **Eva Kappe:** Formal analysis, Data curation, Conceptualization. **Werner Heering:** Methodology, Investigation. **Andreas Brühschwein:** Methodology, Investigation, Formal analysis. **Dörte Lüschow:** Methodology, Investigation, Formal analysis, Data curation. **Ferdinand Schmitt:** Writing – original draft, Investigation, Formal analysis, Data curation, Conceptualization.

## Disclosures

The authors declare that they have no known competing financial interests or personal relationships that could have appeared to influence the work reported in this paper.

## Appendix Supplementary materials

Image, application 1

## Footnotes

Supplementary material associated with this article can be found, in the online version, at doi:10.1016/j.psj.2026.107029.
