# Supplementary material for: Multiple cases of osteopetrosis-like disease in young broiler flocks in Germany
Source: Poult Sci. 2026 Apr 30;105(8):107029. doi: 10.1016/j.psj.2026.107029 (PMC13157146; doi:10.1016/j.psj.2026.107029)
Supplement: Supplementary file 1 [file mmc1.docx]

**Supplementary File.** Results of the antigen ELISA for cloacal samples of osteopetrosis-affected flock in 2023. S/P Ratio: Sample-to-Positive ratio

| Case ID | Sample | O.D. Value | S/P Ratio | Result |
| --- | --- | --- | --- | --- |
| 2023-1453-1 | 1 | 0.171 | 0.212 | Positive |
|  | 2 | 0.336 | 0.498 | Positive |
|  | 3 | 0.108 | 0.102 | Negative |
|  | 4 | 0.215 | 0.288 | Positive |
|  | 5 | 0.060 | 0.019 | Negative |
|  | 6 | 0.070 | 0.036 | Negative |
|  | 7 | 0.063 | 0.024 | Negative |
|  | 8 | 0.079 | 0.052 | Negative |
|  | 9 | 0.075 | 0.045 | Negative |
|  | 10 | 0.058 | 0.016 | Negative |
| 2023-1453-2 | 1 | 0.072 | 0.040 | Negative |
|  | 2 | 0.110 | 0.106 | Negative |
|  | 3 | 0.054 | 0.009 | Negative |
|  | 4 | 0.276 | 0.394 | Positive |
|  | 5 | 0.061 | 0.021 | Negative |
|  | 6 | 0.133 | 0.146 | Negative |
|  | 7 | 0.091 | 0.073 | Negative |
|  | 8 | 0.053 | 0.007 | Negative |
|  | 9 | 0.163 | 0.198 | Negative |
|  | 10 | 0.108 | 0.102 | Negative |
| Total |  |  |  | **4/20 (20%)** |
